# Antibody (Serology) Tests for COVID-19: a Case Study

**DOI:** 10.1128/mSphere.00201-21

**Published:** 2021-05-12

**Authors:** Rachel M. West, Amanda Kobokovich, Nancy Connell, Gigi Kwik Gronvall

**Affiliations:** a Johns Hopkins Center for Health Security, Baltimore, Maryland, USA; b Johns Hopkins Bloomberg School of Public Health, W. Harry Feinstone Department of Molecular Microbiology and Immunology, Baltimore, Maryland, USA; c Johns Hopkins Bloomberg School of Public Health, Department of Environmental Health and Engineering, Baltimore, Maryland, USA; University of Michigan-Ann Arbor

**Keywords:** COVID-19, serology, diagnostics, policy, SARS-CoV-2

## Abstract

Serology (antibody) tests to detect previous SARS-CoV-2 infection have been in high demand from the beginning of the COVID-19 pandemic. The initial shortage of diagnostic tests coupled with asymptomatic infections led to a significant demand for serology tests to identify past infections. Despite serious limitations on the interpretation of a positive antibody test in terms of immunity to SARS-CoV-2, antibody testing was initially considered for release from social distancing, return to employment, and “immunity passports.” The regulatory approach to antibody tests was limited; manufacturers were encouraged to develop and market antibody tests without submitting validation data to the FDA. FDA guidance grew more stringent, but many poor-quality tests were already on the market—potentially inappropriately used for individual decision-making. This is a case study describing COVID-19 serology tests and the U.S. market and describes lessons learned for a future health security crisis.

## Abstract (precis)

Serology (antibody) tests to detect previous SARS-CoV-2 infection have been in high demand from the beginning of the COVID-19 pandemic. The initial shortage of diagnostic tests coupled with asymptomatic infections led to a significant demand for serology tests to identify past infections.

## OPINION/HYPOTHESIS

## AN EXAMPLE OF INSUFFICIENT REGULATION AND HIGH CONSUMER DEMAND

This paper focuses on the development, marketing, and regulation of serology (antibody) tests for COVID-19 in the United States and offers specific recommendations for future health security crises. Serology tests are used to detect patient antibodies specific to SARS-CoV-2, and the presence of anti-SARS-CoV-2 antibodies (seropositivity) can indicate prior infection (1, 2). Serology tests typically measure IgM antibodies specific to the virus, which form 5 to 10 days after initial infection, and/or IgG antibodies, which form 7 to 10 or more days after initial infection (3, 4). Though it is possible that a person who tests positive with a serology test may still be infectious, serology tests should not be used to diagnose current infections (5).

The meaning of SARS-CoV-2 seropositivity for individual patients remains unclear (6). Past infection with SARS-CoV-2 is thought to provide some immunity from COVID-19 disease. However, it remains uncertain how long immunity persists, and what level of antibodies (titer) is sufficient for immunity. Reinfections have occurred (7). It is not recommended for those who are seropositive to exempt themselves from social distancing or mask use, and they are currently recommended to be vaccinated to protect against COVID-19 (8, 9). In the early days of the pandemic, there was much more uncertainty about whether people who had recovered from SARS-CoV-2 were immune, or whether they could be reinfected. Despite the immunological uncertainties, antibody tests were described by political leaders as a tool to “open up the economy” and return to “normal” life—even before such tests were available. There was great demand for the tests from individuals who wanted to know if they had already been infected, perhaps asymptomatically, and could therefore avoid restrictive public health measures. The potential for convalescent plasma for use as a therapy was also dependent on donors who were recovered from COVID-19, often measured by seropositivity (10).

The FDA took steps that were encouraging to manufacturers to produce serology tests. Their initial regulatory approach, however, created a situation where many unvalidated, low-quality tests flooded the market. After 7 weeks, and after Congressional inquiries in response to reports of consumer fraud, FDA regulatory measures were made more stringent. Nonetheless, poor-quality tests remained in use, potentially giving individuals false medical information that could increase their risk of contracting COVID-19. The FDA has granted Emergency Use Authorization (EUA) to 75 SARS-CoV-2 antibody and other adaptive immune response tests as of 13 April 2021. The EUA process, different than a full FDA approval, allows for temporary marketing and use of medical products during a declared public health emergency if there are no “adequate, approved, and available alternatives” to the product, the benefits outweigh the risks, and the product “may be effective” at diagnosing COVID-19 (11).

Early consumer demand for antibody tests was also driven by lack of access to diagnostic testing in the beginning of the pandemic. As recent work has identified that ∼20% of infections are asymptomatic, an antibody test was the only way to identify past infection (12). Our understanding of asymptomatic infections continues to evolve (13, 14). Serology tests continue to be in high demand; consumers pay $40 to $150 out of pocket (15, 16). However, even highly accurate antibody tests yield a percentage of false positives and negatives that complicate individual decision-making, and tests for SARS-CoV-2 antibodies are often not as accurate as advertised (2). In cases where it is important to have an accurate result using a serological test, such as for diagnosis of HIV, sequential tests are performed to confirm seropositivity, a strategy that should be considered if it becomes important to unequivocally determine past SARS-CoV-2 infection (17).

Examining how SARS-CoV-2 antibody tests were regulated and marketed as a case study offers lessons for future infectious disease outbreaks and health security crises. In future, the demand for serology tests and potential for fraud should be anticipated by public health agencies, and the FDA should produce appropriate guidance for developers to limit consumer fraud (18). An important development in the history of SARS-CoV-2 antibody tests was when independent validation of the tests began; such mechanisms to check the accuracy of tests and manufacturers’ claims should be anticipated and implemented more quickly in the future. For tests whose performance is found to be low quality and does not withstand independent validations, the FDA should issue recalls. Finally, future EUA processes should begin by having target thresholds of sensitivity and specificity, concise timelines, and validation data to be provided from the manufacturer. These steps may limit consumer fraud and poor public health decision-making in future public health emergencies.

## TIMELINE OF ANTIBODY TEST DEVELOPMENT AND USE

**FIG 1:**
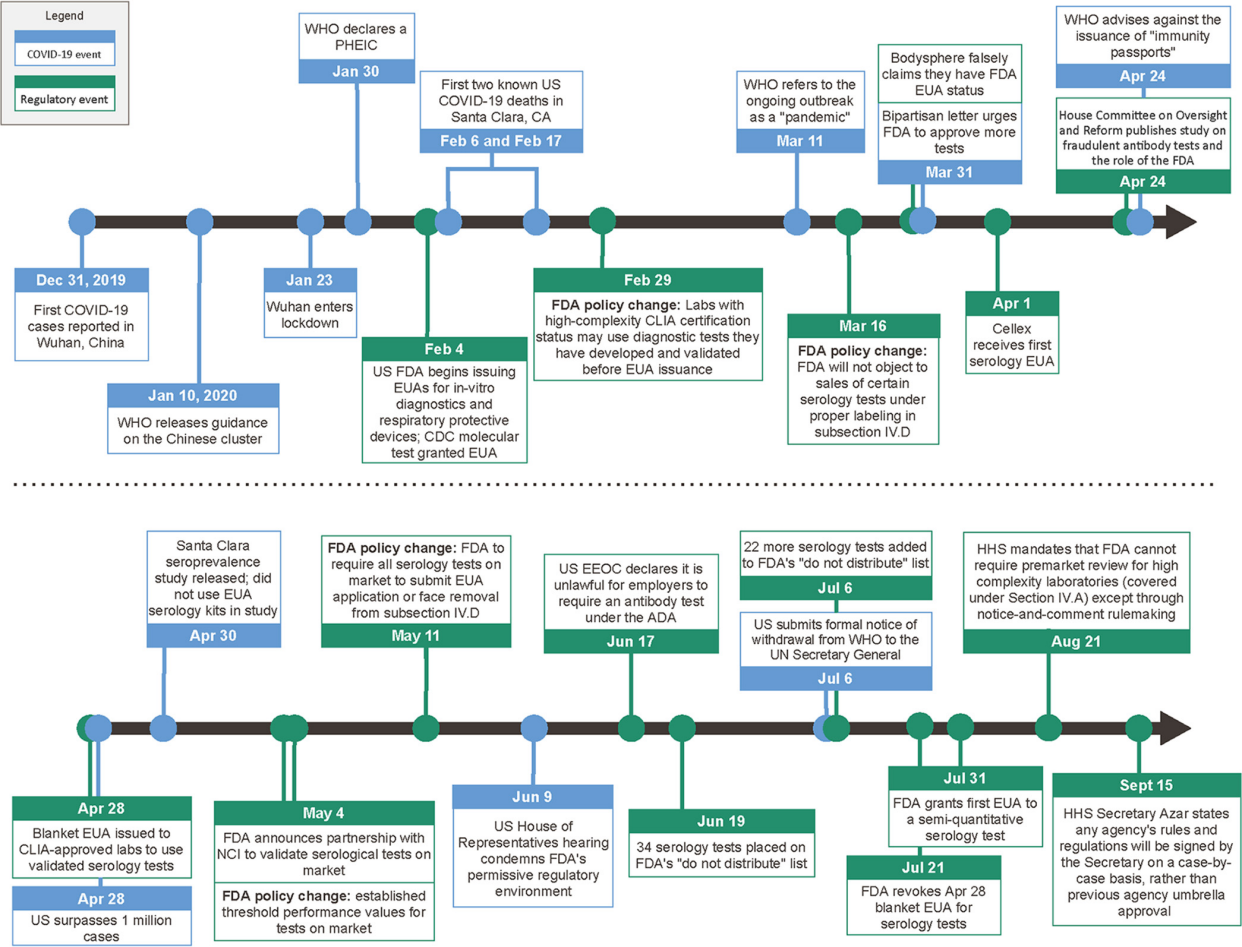
Timeline of the COVID-19 pandemic and U.S. regulatory actions. Events denoted by blue arrows/boxes are specific to the pandemic, and those denoted by green arrows/boxes are U.S. regulatory events. Sources for data are included in the References.

Figure 1 shows the timeline of antibody test development and regulation. The first cases of COVID-19 were reported by China to the World Health Organization on 31 December 2019. The United States detected its first case on 29 February 2020, although the virus had spread before that time (19). Importantly, U.S. public health laboratories were not authorized to diagnose individuals or use tests for SARS-CoV-2 until 29 February and 3 March, respectively (20–22).

**FIG 2:**
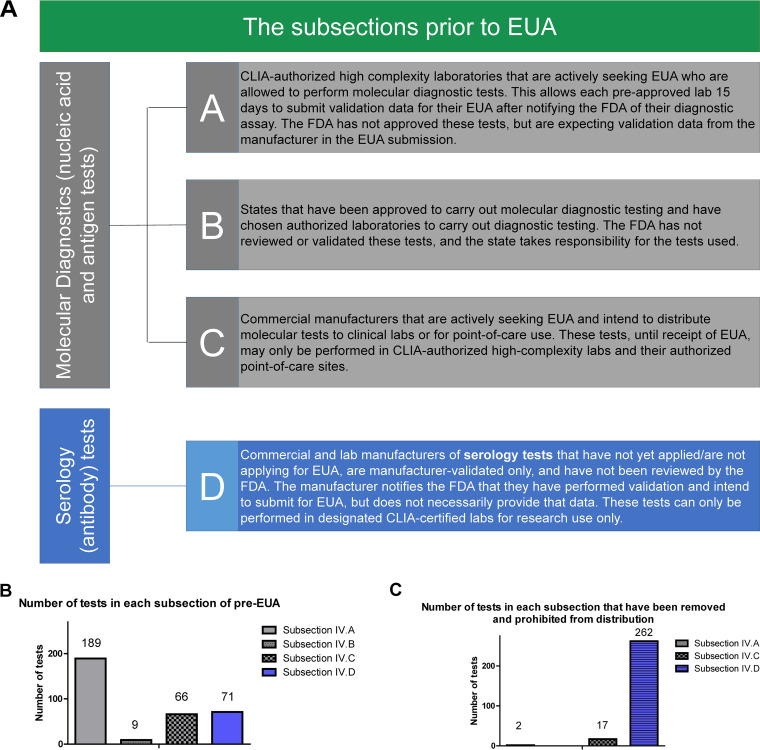
The subsections of pre-FDA Emergency Use Authorization. (A) Manufacturers can market antibody tests through several pathways, described in the FDA Policy on Diagnostic Tests. The FDA stated that tests could be used without submission for EUA in order to encourage test development. No tests within these subsections have received FDA EUA, or FDA approval. As of 11 May 2020, Subsections IV.A through IV.C refer only to molecular/antigen-based diagnostic tests. Subsection IV.D refers only to serology tests (both lab developed and commercial). (B) The number of tests under each subsection at the time of publication. (C) The number of tests under each subsection that have been removed and prohibited from distribution at the time of publication. Sources for data are included in the References.

On 16 March 2020, the FDA issued revised guidance to give test manufacturers 4 regulatory pathways for their tests, described as “subsections” of pre-EUA acknowledgment (Fig. 2A) (23, 24). Rather than requiring manufacturers to submit test validation data to the FDA prior to selling the tests, the FDA stated that it would not “object to the use of validated tests for specimen testing for a reasonable period of time after validation while the laboratory is preparing an EUA request.” Thus, any test manufacturer who notified the FDA could have their product listed on the FDA website under one of these subsections and market their test accordingly. Even though these tests did not have FDA approval or EUA, FDA acknowledgment was confusing to purchasers and gave unvalidated tests inappropriate legitimacy.

**FIG 3:**
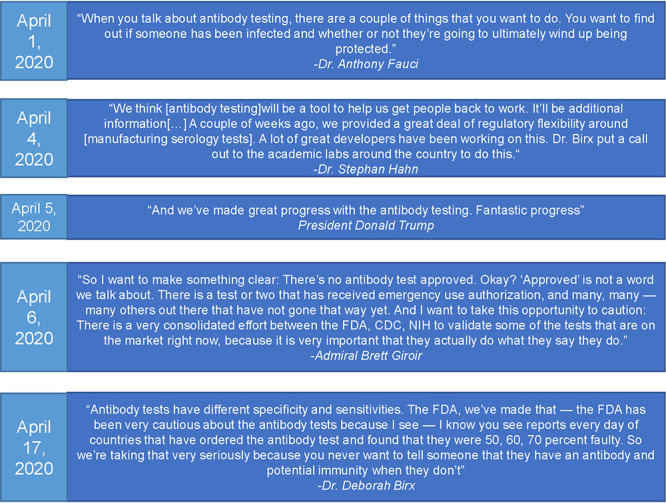
Official language surrounding antibody testing in the United States. Antibody testing was mentioned 45 times during the White House Coronavirus Task Force briefings to the public. Presented here are selected quotes from these briefings. While conflicting information from officials is a product of evolving understanding of the disease, the messaging surrounding serology tests from White House press briefings was inaccurate and may have contributed to the demand for individuals to be tested. All 45 mentions can be found in Table S1.

There was increasing demand for antibody tests from public officials to determine virus prevalence. On 31 March, a bipartisan group of 113 members of Congress urged Alex Azar, Health and Human Services (HHS) Secretary, and Rick Bright, the Director of the Biomedical Advanced Research and Development Authority (BARDA), to rapidly deploy antibody tests (25). “It will be an unnecessary economic tragedy if our citizens remain cowering at home because we failed to provide them with the simple, inexpensive means of proving their immunity,” they wrote. During daily White House press briefings, antibodies and serology testing were mentioned frequently, and not always reflective of the uncertainties about immunity to COVID-19, leading to public and political confusion (Fig. 3; see also Table S1 in the supplemental material).

10.1128/mSphere.00201-21.1 TABLE S1 All mentions of antibody testing during the Trump White House Coronavirus Task Force briefings to the public. Download Table S1, DOCX file, 0.03 MB. Copyright © 2021 West et al. 2021 West et al. https://creativecommons.org/licenses/by/4.0/ This content is distributed under the terms of the Creative Commons Attribution 4.0 International license.

Following the decision to allow product marketing without an EUA, over 175 serology tests entered the market, in what was described as a “Wild West” (26). Well-established companies joined lesser-known health companies that marketed scar-minimizing lotions or “male enhancement powder” in marketing serology tests (27, 28) One manufacturer, Bodysphere, featured scantily clad women on its website and exotic cats for its employee profile pictures. The company falsely declared it had an EUA for its serology test, which was explicitly refuted by the FDA on 31 March, though not before the test was reported as a breakthrough (29, 30). Other manufacturers continued to claim FDA approval despite having Subsection IV.D status (i.e., no FDA review) (Fig. 2B and C).

Antibody tests were also used for public health serosurveys, to determine the extent of disease spread, the true case fatality rate, and whether public health mitigation measures were effective (31). After a New York seroprevalence study found significant asymptomatic spread, demand for antibody tests climbed, as a positive test was falsely equated with immunity and release from physical distancing (32). LabCorp began offering tests to the public, with a prescription (33). California testing facilities were inundated by those who believed they had COVID-19 before testing was available (34). Researchers scrambled to develop tests quickly that could be deployed locally (35).

With restrictive public health measures reshaping society, “immunity passports” or “certificates” based on seropositivity were considered in order to reopen the economy and allow travel. Germany was an early proponent for exempting those who were seropositive from restrictive physical distancing measures (36). Chile, the United Kingdom, and the United States followed (37, 38). Companies formed to market digital immunity passports (e.g., COVI-PASS digital health wallet). Scientists and public health experts objected based on the scientific uncertainty of immunity to COVID and on concerns that immunity passports would create perverse incentives leading people to try to become infected (6, 40, 41). Immunity to SARS-CoV-2 was, and is, incompletely understood—the necessary levels of antibodies for protection, how long they last, and whether reinfection was possible all limited the potential for immunity certificates. By the end of April, the WHO had issued a warning against immunity passports, among calls by bioethicists for ending the practice (42).

On 1 April, the FDA granted the first serology test EUA to Cellex Inc., for a lateral flow assay that detected IgM and IgG in the patient serum, giving a qualitative “positive/negative” readout (43). However, states continued to purchase tests without EUA. In one incident, the city of Laredo, TX, spent $500,000 to purchase tests, in the mistaken belief they would be helpful for diagnosing active infections (44). Instead of the manufacturer-advertised 97% specificity, the test specificity was close to 20%. Reports of poor-quality tests occurred throughout the world, including in the United Kingdom, where millions of tests were found to be unusable after Oxford University tested them (45). Spain purchased hundreds of thousands of faulty tests, and after the first defective batch was returned, the second batch was also found to be defective (46). Slovakia bought millions of ineffective antibody tests, and Denmark returned over a million faulty underperforming tests (47, 48).

The House Committee on Oversight and Reform, Subcommittee on Economic and Consumer Policy, chaired by Rep. Raja Krishnamoorthi, sent a letter to the FDA Commissioner on 9 April, concerned about consumer fraud in antibody testing. Their preliminary investigation found the FDA had allowed serology tests to be marketed and sold without review, leading to “flawed” White House plans to reopen the country (49). When manufacturers were contacted by the committee, requests were ignored. The report stated there was no enforcement by the FDA to ensure that fraudulent tests were removed from the market, and it identified a lack of gold-standard testing against which new serology tests could be easily compared. A lack of validation data for EUA-granted tests was still a problem, with results from only 2 EUA-granted tests available to the public (50).

On 28 April, the FDA authorized a blanket EUA for serology tests developed by authorized laboratories under the Clinical Laboratory Improvement Amendments of 1988 (CLIA), 42 U.S.C. §263a (51). This allowed certified labs to develop and utilize serology tests and potentially have them independently validated by the National Cancer Institute (NCI), without a full EUA application. The CDC serology test protocol fell under this umbrella EUA initially (52).

On 4 May, the FDA produced a stricter policy for serology tests and a plan for validation (53). It required thresholds for sensitivity and specificity of tests that were eligible to apply for EUA (54). These thresholds included testing at least 30 positive and 75 negative samples and required a sensitivity and specificity of at least 90% and 95%, respectively, with a required IgM sensitivity of 70% and an IgG sensitivity of 90%. The new policy also required manufacturers to provide validation data to the FDA within 10 days after notification of submission for EUA. At that time, tests under the umbrella EUA were required to be independently validated (51). For validation, the FDA partnered with the National Cancer Institute (NCI) due to their extensive capacity for serology testing (55). One week later, the FDA released templates for submitting for EUA for commercial and lab-developed serology tests (54, 56). These provided clear directions for any manufacturer for the amount and types of data required for submitting EUA paperwork.

Validation studies performed by the NCI led to immediate changes in the market, including the removal of Chembio’s poorly performing test from the EUA list on 16 June (57, 58). However, not all of the tests with EUA have both the manufacturer and NCI validation data publicly available (59). Several serology tests which since have received independent validation include such information within their instructions for use (IFUs); while this is not mandatory or true of all tests, those that choose to include this provide greater transparency for the consumer (60, 61). More recent updates to this validation process have prioritized validation of lateral flow assays and enzyme-linked immunosorbent assays (ELISAs) by the NCI and NIH (62).

The FDA began to issue warning letters to manufacturers who inaccurately marketed their tests as FDA approved on 16 June (Fig. 2C) (63, 64). The FDA also requested that members of the public report any fraudulent test sales to the health fraud site (65). Although sales of inaccurate tests were prohibited, there were no recalls on products already sold and distributed.

On 21 July, the FDA chose to revoke the umbrella EUA for serology tests (66). The FDA stated that individual EUAs would give the agency greater insight and control over authorized tests and chose to revoke the umbrella EUA. Of the tests under the initial umbrella EUA, none had been included in Subsection IV.A or granted EUA at the time of revocation.

By August, 40 commercial serology tests had an EUA. Proposed uses of antibody tests also began to shift. The CDC recommended that serology tests “can be offered as a method to support diagnosis of acute COVID-19 illness for persons who present late” (67). They clarified that serology tests should not be used to determine immune status and that care should be taken in low-prevalence areas to acknowledge the positive predictive value of the tests. A recent meeting of over 300 scientists resulted in publication of recommendations for next steps, namely, to increase available antibody diagnostics, independently validate them, and use them to distinguish vaccine-induced and naturally occurring immune responses (68).

The Department of Health and Human Services implemented another policy change regarding the FDA on 21 August: the FDA does not have authority to grant EUAs to high-complexity laboratories (usually covered under Section IV.A) (69). Calling such rules “duplicative regulations” that would interfere with the COVID-19 response, HHS issued the rescission that essentially removes premarket review of laboratory-developed tests (LDTs) (70). High-complexity labs are highly variable in size and capacity and thus not equally equipped to develop and run tests. Removal of EUA oversight may not have a significant impact on serology test availability, as bottlenecks have occurred with commercial tests, not LDTs (71). However, it will reduce the ability of the FDA to review LDT protocols of smaller, less established labs that may struggle to meet testing demands. Former FDA Deputy Chief of Staff Kalah Auchincloss expressed concerns about reflooding the market with faulty serology tests, arguing that “the thought that this is somehow going to spur innovation while not compromising safety is just absurd” (72). On 20 August, Rep. Frank Pallone, chairman of the Committee on Energy and Commerce, demanded an explanation from HHS Secretary Azar, concerned that “flooding the market with unregulated and potentially inaccurate tests will only further undermine our nation’s response efforts.” (73). The FDA policy change was speculated to be a political response to the halt on the FDA EUA for convalescent plasma treatments due to a lack of strong evidence for efficacy, an action condemned by former president Donald Trump (74).

In November 2020, the FDA issued further templates for manufacturers of serology sample collection kits that use fingerstick dried blood spots under prescription use (75). Such kits could allow easy sample collection for further analysis with authorized tests. The templates included human usability study requirements, as well as minimum sample requirements for clinical performance validation.

In March of 2021, serology testing was expanded to include “other adaptive immune response” tests, including those which measure T-cell responses and neutralizing antibody responses. FDA templates were accordingly updated (76, 77). These templates also address the potential complications presented by emerging variants of SARS-CoV-2 and suggest that manufacturers monitor how mutations may impact their test. The revisions also include slightly lower thresholds—for instance, a combined IgM/IgG sensitivity of 87% compared to 90%—of positive percent agreement and negative percent agreement if sample sizes are sufficiently large. With the advent of vaccines, new EUAs often have language stating that serology tests results should not be “interpreted as an indication or degree of protection from infection after vaccinations” (78). No serology tests with EUA to date have been validated on sera from vaccinated individuals.

## RECOMMENDATIONS

The tumultuous history of serology testing in the United States over the course of the COVID-19 pandemic offers lessons about medical testing development in an emerging epidemic, consumer demand and choice in a politicized environment, and shortfalls in our current regulatory environment. These issues should be considered when preparing for\a future health security crisis.

### The FDA should anticipate consumer demand for tests and take steps to prevent consumer fraud.

There was considerable demand for serology tests. One study showed that 80% of the public would want an antibody test if available at little to no cost (79). The perception that a positive antibody test could allow for a safe return to work, travel, or social events likely motivated testing (80). The public demand, however, makes the plethora of low-quality tests that much riskier, particularly if after testing individuals acted counter to public health guidance. Beyond COVID-19, in an age of personalized medicine and at-home ancestry kits, people are likely to continue to expect quantitative health information delivered quickly, and this consumer demand should be anticipated in the future. The FDA and other public health agencies should learn from this pandemic’s increased demand for quality tests and take steps to prepare clear testing authorization processes. This could include continued communication with the public on any testing policy updates, avenues for reporting tests with fraudulent claims of FDA approval, and engaging with manufacturers on preparing EUA applications.

### There should not be a Subsection IV.D status prior to Emergency Use Authorization.

Given the potential for consumer fraud, there should not be a “Section IV.D” wherein FDA neither reviews the tests, receives validation data, nor requires accuracy thresholds or certification of laboratories. The terms of this Subsection led to a flood of poor-quality tests that had the public veneer of FDA acceptability, as they were listed on FDA web pages. To date, 240 serology tests have been prohibited from distribution from this Subsection IV.D list. Further, language should be exceptionally clear regarding approval status, clarifying the difference between EUA and full approval.

### The FDA should collaborate with qualified labs and organizations to independently validate tests during a public health emergency.

Independent efforts to validate serology test performance are essential to understand the quality, i.e., the sensitivity and specificity, of novel tests. Early validation efforts were led by academic and medical research institutions, comparing emerging serology tests on the market (81). Independent and national efforts in the United Kingdom, France, and China early in the pandemic highlighted the best-performing serology tests (82–84). After 2 months of relying on manufacturer claims, the FDA began a partnership with the NCI to independently validate tests submitted for EUA. This delay led to low-quality tests being submitted for EUA and remaining in Subsection IV.D, as well as tests receiving EUA that were later rescinded. The requirement for independent validation to receive EUA from the beginning could have prevented a faulty test from entering the market. Independent validation studies may be required if tests begin to measure clinical performance on sera from vaccinated individuals. A similar panel of sera from vaccinated individuals would be beneficial in validating any claims that manufacturers state regarding test performance in this population. Funding for independent validation could be provided by government pandemic funding, such as through BARDA, and should be considered for future pandemic preparedness planning and funding (85, 86). Reference samples may not be immediately available at the beginning of a pandemic, but planning to collaborate with agencies such as the CDC would allow more efficient sample access. Collaborations for independent validation studies should exist even before pandemics. Further, these data should also be made transparent; validation information appeared in product inserts but was not clear on the agency websites. Updates to the NCI collaboration and validation efforts have provided greater transparency of test performance, though inclusion of the results of validation studies in manufacturer IFUs is not yet mandatory. Those manufacturers that include the independent validation studies are commendable, in that they provide greater clarity and lend their test credibility.

### The FDA should recall poor-quality tests from the market.

The FDA issued multiple warning letters to companies fraudulently claiming FDA approval and posted lists of tests prohibited from distribution on their FAQ website (64, 87). They have also revoked EUA from 2 serology tests, Autobio and Chembio, for poor performance during independent testing (57, 88). However, there have been no recalls. The FDA recommends that health care providers stop using these tests but does not require it (89). A recall could help to ensure that poor-quality tests are not sold by third parties or accidentally used in a health care or research setting. Future EUA programs should implement a recall mechanism similar to existing FDA recall methods with medical and food products (90). If a recall mechanism is not feasible, the FDA should aim to communicate clearly and often with health care providers to give relevant information on low-quality tests. While the FDA recommended that providers check the FDA website for such tests, a weekly update could be useful for health care workers to stay up to date on revoked EUAs and warnings (64). These revoked EUAs have already caused financial issues, with a case involving Virginia Tech suing for reimbursement (91).

### Future EUA approval processes should begin by having required thresholds of sensitivity and specificity, concise timelines, and validation data from the manufacturer.

Currently, the EUA template requires 30 positive and 75 negative clinical samples for determination of sensitivity and specificity, respectively. This should be significantly increased (to at least 100 samples each, for example) to better represent diversity of samples and consistency of the test. Sensitivity and specificity must be high, but in a low-prevalence disease setting (such as the onset of an outbreak), this requires a large sample size (92). Ideally, the sample size in a low-prevalence setting for a high statistical power (above 80%) and very high sensitivity and specificity thresholds should be in the hundreds. However, if samples are difficult to obtain, the sample size should be as large as possible and increase as samples become more widely available. These sample sizes can be estimated with software such as PASS (93). In addition, language in the template should clarify that these are true clinical samples, not contrived samples with antibodies spiked into the serum at a known concentration. More recent updates to the EUA templates have gone into greater detail regarding minimum sample size, sample stratification based on time from symptom onset, and application of the serology test. These templates have also included novel sample collection and analysis methods, which is a proactive measure for manufacturers seeking to market these tests. Continuous updates to FDA documents, such as these templates, are essential to ensure that regulations evolve alongside testing capabilities. Independent validation studies in the future may consider including sera from vaccinated individuals to examine test performance, though these types of EUAs have not yet been issued.

These performance thresholds should be coupled with a preapproved, gold-standard diagnostic. This gold-standard test would be the test against which novel diagnostics are measured. Having a consistent standard will make the performance results of tests more generalizable and give manufacturers a clear protocol for validation. An example of this is the NCI’s panel of positive and negative samples used to validate EUA tests. Because these samples are consistent, it is easier to compare 2 novel tests to one another. The gold standard of determining test positivity may be a molecular test in early stages of an outbreak. But as serology tests are continuously validated over time, it would be beneficial to have a gold-standard serology test against which performance can be measured. While positive predictive values, and other measures of test performance, can change with disease prevalence, preliminary thresholds for sensitivity and specificity could be established.

## CONCLUSION

There are opportunities to learn from the COVID-19 pandemic, even as it persists. The FDA’s regulatory processes evolved alongside the pandemic, and the improvements they have implemented can serve as lessons for future health security crises. Recent commentaries have indicated that the FDA is willing and ready to use its experience to improve future responses (18). Serology test developers should have guidance and thresholds to promote quality test development. Independent validation of EUA tests would ensure quality test performance. The FDA must also anticipate the public and political demand for serology tests moving forward. As the COVID-19 pandemic continues, the FDA must stand as a source of evidence-based guidance to protect public health.
