# Supplementary material for: Antibody (Serology) Tests for COVID-19: a Case Study
Source: mSphere. 2021 May 12;6(3):e00201-21. doi: 10.1128/mSphere.00201-21 (PMC8125050; doi:10.1128/mSphere.00201-21)
Supplement: TABLE S1 [file mSphere.00201-21-st001.docx]

| **Date of Press Briefing** | **Link** | **Number of comments on antibody tests** | **Speaker** | **Quote** | **Nearest Date of FDA Policy Change on Serology EUAs** |
| --- | --- | --- | --- | --- | --- |
| 3/23/2020 | https://www.c-span.org/video/?470599-1/president-trump-coronavirus-task-force-hold-briefing-white-house | 2 | Dr. Deborah Birx | Q: Dr. Birx, when will the government roll out the antibody tests so people can know possibly if they’ve been exposed? DR. BIRX: So all — several of them have come to the FDA, I believe. Obviously that’s something I am very interested in for two reasons. One, it will give us a retrospective on where these infections were, who was actually infected, and how really asymptomatic versus mild versus all of that comes into the spectrum. Secondly, by people who have high titers of those antibodies, those can become our solution with plasma freezes for those in need and the making of hyperimmune globulin. So knowing who they are becomes really critical. But I think we’re still a couple of weeks out. | 3/16/2020 |
|  |  |  |  | Q:And, Dr. Birx, a question about the serology blood tests, which some people said hold some real promise here. When will those be FDA approved and when would they be widely available? DR. BIRX: So that’s what she was just asking about. So these IgG and IgM — both point of care — I can tell you it would be no problem making ELISA today. But then you’re drawing blood, you’re processing blood, you’re running plates; that’s what we did in the past. We really want a finger-prick-type assay where you can just put a fingerprint on and get your IgG and IgM. So that’s what we’re working on right now. |  |
| 4/1/2020 | https://www.c-span.org/video/?470864-1/white-house-announces-counter-narcotics-operation-pacific-ocean-caribbean | 7 | Dr. Deborah Birx | And I think really being able to tell them — the peace of mind that would come from knowing you already were infected, you have antibody, you’re safe from reinfection, 99.9 percent of the time |  |
|  |  |  |  | I’ve talked to a lot of them over the last few days to really ask them to develop these simple ELISA tests that could be used rapidly in their healthcare centers. Because immediately, with a — it’s easy to do; we’ve all developed ELISAs — so in a day or two after development, they could screen their entire hospital. |  |
|  |  |  |  | And so I really called on every university in every state to develop ELISAs. |  |
|  |  |  |  | It[developing ELISAs] could happen soon, within this month, if the universities help us. Absolutely. |  |
|  |  |  | Dr. Anthony Fauci | Okay. So when you talk about antibody testing, there are a couple of things that you want to do. You want to find out if someone has been infected and whether or not they’re going to ultimately wind up being protected. |  |
|  |  |  |  | Antibody testing right now is not the first thing on our priority. |  |
|  |  |  |  | And that is: Back in the day, when [Dr. Birx] and I were both doing the HIV/AIDS issues back when we first discovered the virus in ‘83 and we had an antibody test in 1985, we found out by SIRO surveillance representative in different populations that we were dealing with the tip of the iceberg when we saw individuals who were the ones who actually got infected. It gave us a really good feel for how many people are infected, how many are doing well, and how many are getting ill. |  |
| 4/4/2020 | https://www.c-span.org/video/?470970-1/president-trump-comments-firing-intelligence-community-inspector-general | 2 | Dr. Stephen Hahn | We think [antibody testing]will be a tool to help us get people back to work. It’ll be additional information. Because, as you know, if you have an antibody, that means you were exposed and have recovered from it. |  |
|  |  |  |  | Q: But how quickly can you scale up this testing to determine on a large scale how many people can go back to work and have this antibodies? DR. HAHN: So, as you know, a couple weeks ago, we provided a great deal of regulatory flexibility around this. A lot of great developers have been working on this. Dr. Birx put a call out to the academic labs around the country to do this. And we’ve been working very closely with a number of manufacturers. So we think that it can be scaled up relatively quickly. |  |
| 4/5/2020 | https://www.c-span.org/video/?470975-1/president-trump-coronavirus-task-force-briefing | 3 | Dr. Anthony Fauci | And trust me, that [the number of asymptomatic cases] is a estimate. I don’t have any scientific data yet to say that. You know when we’ll get the scientific data, when we get those antibody tests out there and we really know what the penetrance is. |  |
|  |  |  | Donald Trump | And we’ve made great progress with the antibody testing. Fantastic progress |  |
|  |  |  | Dr. Deborah Birx | And just to tell you how we’re kind of doing that: For New York, which you can see at the top, their serology is now about 36 percent positive. |  |
| 4/6/2020 | https://www.c-span.org/video/?470990-1/president-trump-coronavirus-task-force-briefing&playEvent | 7 | Admiral Brett Giroir | As opposed to the test for a novel virus, the antibody-type tests are very sophisticated technology, but they’re old technology. This, we expect to have many tens of millions of tests the first month that we are really sure that the test makes sense |  |
|  |  |  |  | So — so we’re — science doesn’t run on rails. Right? So we need to make sure that the FDA, the NIH — which they’re actively doing right now — assure that the [antibody] tests that they’re testing really do perform the way they should. |  |
|  |  |  | Dr. Deborah Birx | So I just want to assure the American people that as we’re working on the crisis of today, we’re looking to the future to ensure we have the systems in place to bring those early alert systems together while we work on the antibody testing and while we work on continuing, spreading, and increasing the diagnostic capabilities that you see presented here. |  |
|  |  |  | Dr. Anthony Fauci | And that’s the reason why — it was discussed up here — why it’s so important to have an antibody test so you know what the penetrance is in society. |  |
|  |  |  | Donald Trump | Q:Mr. President, on the question of antibody testing — which is FDA approved now, but not widely available yet — I know the Admiral said, by May, expect to have millions available. How are you going to prioritize who’s going to get the antibody tests? And what does — what is that going to show you? Do you think that’s going to be immunity? THE PRESIDENT: Okay. I mean, I have an answer, but I’d rather have the Admiral answer that. |  |
|  |  |  | Admiral Brett Giroir | So, let me clarify — and I know you probably understand this — is the antibody test does not tell you if you have the active virus in your nose. If you’re positive for the antibody, it strongly implies — it means that you have had the virus before. And to the degree that we know of medical knowledge, you will probably — highly probably — be protected against getting the virus again in the future. |  |
|  |  |  |  | So I want to make something clear: There’s no antibody test approved. Okay? “Approved” is not a word we talk about. There is a test or two that has received emergency use authorization, and many, many — many others out there that have not gone that way yet. And I want to take this opportunity to caution: There is a very consolidated effort between the FDA, CDC, NIH to validate some of the tests that are on the market right now, because it is very important that they actually do what they say they do. |  |
|  |  |  |  | And we have reason to believe that not all of [the antibody tests] are going to perform well. I don’t know the primary source, but the Financial Times just reported that the UK had 17.5 million antibody tests that they bought, and none of them work. So we’re not going to get in that situation. |  |
| 4/17/2020 | https://www.c-span.org/video/?471279-1/president-trump-coronavirus-task-force-briefing | 7 | Donald Trump | Earlier this week, the FDA authorized two new antibody tests — which is very exciting — that will determine if someone has been previously infected with the virus, bringing the total to four authorized antibody tests already. This will help us assess the number of cases that have been asymptomatic or mildly symptomatic, and support our efforts to get Americans back to work by showing us who might have developed the wonderful, beautiful immunity |  |
|  |  |  | Mike Pence | We all know about the 15-minute Abbott test, but the FDA is currently working on an antibody test that literally could add 20 million new tests to our supply, even before the end of April. |  |
|  |  |  | Dr. Anthony Fauci | Now, the other test is an antibody test — a test that tells you, in fact, that you’ve been infected. That’s really good. |  |
|  |  |  |  | I mean, we are assuming that if you’re infected and you have antibody, you’re protected. And I think that’s a reasonable assumption, based on our experience with other viruses. |  |
|  |  |  | Dr. Deborah Birx | I want to just leave you with my last concept on the antibody tests. Antibody tests have different specificity and sensitivities. The FDA, we’ve made that — the FDA has been very cautious about the antibody tests because I see — I know you see reports every day of countries that have ordered the antibody test and found that they were 50, 60, 70 percent faulty. So we’re taking that very seriously because you never want to tell someone that they have an antibody and potential immunity when they don’t. And so those tests perform better when there’s a high prevalence or a high incidence of disease. |  |
|  |  |  |  | Because I think you’ll see, as more and more articles come out for surveillance that other — and monitoring that other states have done, higher and higher antibody in multiple individuals who don’t remember having a sickness. And that will give us an idea — that’s our asymptomatic monitoring in these sentinel monitoring sites. |  |
|  |  |  |  | So we want to work with mayors around the United States, as those antibody tests become available, to really see what it is in first responders and healthcare workers in the highest prevalence states, so that we can know about the quality and the real-life, real-field experience of those assays. |  |
| 4/22/2020 | https://www.c-span.org/video/?471421-1/president-trump-voices-disagreement-georgia-governor-reopening-plan | 1 | Dr. Deborah Birx | But in parallel, we’re working with states and local governments to really define what that population is by doing — in collaboration with states, working with them together, to really reinforce this antibody testing but in a careful way where you do two antibody testing to increase your sensitivity and specificity into the 99-plus percent range, because we think it’s really important that you have a very high-quality test, but a high-quality test that you can really tell someone that they’ve had this before. |  |
| 4/24/2020 | https://www.c-span.org/video/?471479-1/president-trump-coronavirus-task-force-briefing | 5 | Dr. Stephen Hahn | I updated you earlier this week on serologic tests — these antibody tests that are used to detect natural immunity — and the FDA’s approach to help make these tests available. While these are just one part of our larger response effort, they can play a role in helping move our economy forward by helping healthcare professionals identify those who have immunity to the COVID-19 |  |
|  |  |  |  | To date, under our emergency use authorization approach, we’ve quickly reviewed and authorized 63 tests, both diagnostic, as well as serologic — that is the antibody tests. |  |
|  |  |  |  | Q — and it’s timely, because just about an hour ago, a Subcommittee with Oversight released some findings that the FDA doesn’t have any review of the antibody tests that are on the market. There are no guidelines to tell which ones should be out there, and there’s no way to test their accuracy. They’re quite worried that these are junk tests on the market because they weren’t reviewed before they were approved. Is that true? DR. HAHN: So, under our policy, we provide a flexibility. What we’ve told manufacturers is that, in order to market in the U.S., they have to validate their tests, they have to tell us that they validated their tests, and then, in the package insert, they have to let people know — end users, labs, et cetera — that those tests were not authorized by FDA. |  |
|  |  |  |  | We’ve authorized four [serology tests]. As I mentioned, more are in the pipeline. And these tests that have come in without any information to us, but have been self-validated — as I mentioned at the podium a couple days ago, we are working with the National Cancer Institute, as well as CDC, to perform our own validation of the tests that that have been sent to us. So we’ll provide as much information as we possibly can. And there is transparency on our website about those tests, and also the tests that we have authorized. |  |
|  |  |  | Mike Pence | Minnesota Governor Tim Walz reflected on the call today about the partnership he’s forged with the Mayo Clinic, the University of Minnesota, and state health department. They’re actually collaborating to perform 20,000 molecular tests and 15,000 antibody tests per day. |  |
| 5/5/2020 | https://www.c-span.org/video/?c4873921/president-trump-coronavirus-task-force | 1 | Donald Trump | And I just — I just want to say that we’ve done an incredible job on testing. With that being said, we have some additional, including antibody tests, coming out that will even blow these numbers away. But nobody has done the job we’ve done. |  |
| 5/6/2020 | https://www.c-span.org/video/?471841-1/white-house-coronavirus-task-force-stay | 5 | Dr. Caitlin Pedati | Serology tests help us understand patterns of who might be exposed and how we might predict where the virus is moving in Iowa, and how we can direct community resources and get ahead by providing preventive public health support to businesses and healthcare facilities in areas where we think we might see increased activity. | 5/4/2020 |
|  |  |  |  | We know we have much to learn, for example, about serology and whether there is presence of neutralizing antibodies or how long people might enjoy that immunity. |  |
|  |  |  |  | So we began to offer serologic testing in cohorts across the state to help us understand patterns of who might be exposed and how we might predict where the virus is moving in Iowa, and how we can direct community resources and get ahead by providing preventive public health support to businesses and healthcare facilities in areas where we think we might see increased activity. |  |
|  |  |  | Governor Kim Reynolds | I think, though, doing the serology testing with the diagnostic and PCR, it’s really — there’s — we’re learning more and more about asymptomatic individuals. And so that’s helped us kind of identify that, especially as we’re trying to get the workforce back into the manufacturing and processing plants so that we can keep them up and going. |  |
|  |  |  | Donald Trump | So the media likes to say we have the most cases, but we do, by far, the most testing. If we did very little testing, we wouldn’t have the most cases. So, in a way, by doing all of this testing, we make ourselves look bad. |  |
| 6/22/2020 | https://www.c-span.org/video/?473299-1/press-secretary-president-directed-officials-slow-coronavirus-testing | 1 | Kayleigh McEnany | In fact, the FDA has authorized 144 tests under EUAs — Emergency Use Authorizations. These include 122 molecular tests, 21 antibody tests, one antigen test. And the President has led the way in making us the world’s leader when it — with regard to testing | 5/11/2020 |
| 7/8/2020 | https://www.c-span.org/video/?473708-1/vice-president-reiterates-presidents-call-open-schools-fall | 2 | Dr. Deborah Birx | Those are all good questions, and I think it really comes to the evidence base of what do we have as far as testing and children. So if you look across all of the tests that we’ve done, and whether — when we have the age, the portion that has been the lowest-tested portion is the under-10- year-olds. So we’re putting into place other ways to get testing results from them and looking at antibody in that discarded samples, and try to really figure this out, because parents have really done an amazing job of protecting their children |  |
|  |  |  |  | So all of those [types of tests] are being worked on, and it’s why we’ve been pushing on the antigen test. I know you’ve heard me talk about that in April. We’re pushing on that because we think it is important for testing of students and testing in universities. But we have the — our data is skewed originally to people with symptoms, and then skewed to adults over 18. And so we are looking very closely into that category by using our antibody tests. |  |
| 7/31/2020 | https://www.c-span.org/video/?474416-1/white-house-blames-democrats-lapse-unemployment-benefits | 1 | Kayleigh McEnany | And on top of the more than 59 million tests that we’ve done, the FDA has authorized more than 193 tests under emergency use authorizations, including molecular and antibody tests. | 21-Jul-20 |
| 08/04/2020 | https://www.c-span.org/video/?474531-1/president-trump-comments-covid-19-mortality-rate-calls-lebanon-explosion-attack | 1 | Donald Trump | Last week, the FDA also authorized the first two tests that display an estimated quantity of antibodies present in the individual’s blood, which is a big deal, allowing us to learn more about the immune response. |  |
